# Biomechanics of tendrils and adhesive pads of the climbing passion flower *Passiflora discophora*

**DOI:** 10.1093/jxb/erab456

**Published:** 2021-10-21

**Authors:** Frederike Klimm, Stefanie Schmier, Holger F Bohn, Svenja Kleiser, Marc Thielen, Thomas Speck

**Affiliations:** 1 Plant Biomechanics Group @ Botanic Garden, University of Freiburg, Freiburg, Germany; 2 Freiburg Center for Interactive Materials and Bioinspired Technologies (FIT), Freiburg, Germany; 3 Freiburg Materials Research Center (FMF), Freiburg, Germany; 4 Cluster of Excellence livMatS @ FIT–Freiburg Center for Interactive Materials and Bioinspired Technologies, University of Freiburg, Germany; 5 McGill University, Canada

**Keywords:** Attachment pad, biomechanics, functional morphology, liana, *Passiflora discophora*, tendril

## Abstract

The climbing passion flower *Passiflora discophora* features branched tendrils with multiple adhesive pads at their tips allowing it to attach to large-diameter supports and to flat surfaces. We conducted tensile tests to quantify the performance of this attachment system. We found that the force at failure varies with substrate, ontogenetic state (turgescent or senescent), and tendril size (i.e. tendril cross-sectional area and pad area). The tendrils proved to be well balanced in size and to attach firmly to a variety of substrates (force at failure up to 2N). Pull-off tests performed with tendrils grown on either epoxy, plywood, or beech bark revealed that senescent tendrils could still bear 24, 64, or 100% of the force measured for turgescent tendrils, respectively, thus providing long-lasting attachment at minimal physiological costs. The tendril main axis was typically the weakest part of the adhesive system, whereas the pad–substrate interface never failed. This suggests that the plants use the slight oversizing of adhesive pads as a strategy to cope with ‘unpredictable’ substrates. The pads, together with the spring-like main axis, which can, as shown, dissipate a large amount of energy when straightened, thus constitute a fail-safe attachment system.

## Teaser

Energy-dissipating tendrils of the climbing plant *Passiflora discophora*feature multiple adhesive pads that provide secure and long-lasting attachment to a variety of substrates.

## Introduction

Instead of investing material in a solid self-supporting trunk, climbing plants use other plants or inanimate structures as supports in order to grow upwards (e.g. Darwin, 1882; Isnard and Silk, 2009; Vaughn and Bowling, 2011; Rowe and Speck, 2015). The strength of the attachment generated by the highly diverse attachment structures includes in increasing order: leaf roughness, reflexed leaflets, leaf interlocking, petiole base angles, adventitious roots with root hairs, hooks, tendril organs, sensitive branch angles, sensitive twining branches, and twining main stems (Rowe and Speck, 2015).

Tendrils as defined by Darwin (1882) are filamentary organs that are sensitive to contact and that are used exclusively for climbing. The ontogenetic origins and growth patterns of tendrils vary broadly, as does their shape (Sousa-Baena *et al.*, 2018; Sperotto *et al.*, 2020). Most tendrils attach to their substrate via so-called contact coiling; that is, in response to a mechanical stimulus, the tendril coils around a support (Jaffe and Galston, 1968). Even though the sensitive part of the tendril can be stimulated by extremely small mechanical loads, tendrils do not coil in reaction to wind or rain (Darwin, 1882; Pfeffer, 1885). It is assumed that no lower size limit exists for a support which a tendril can ‘grasp’; to the upper end, however, the diameter of potential supports is limited by the length of the tendril (MacDougal, 1893; Putz and Holbrook, 1991).

**Fig. 1.**
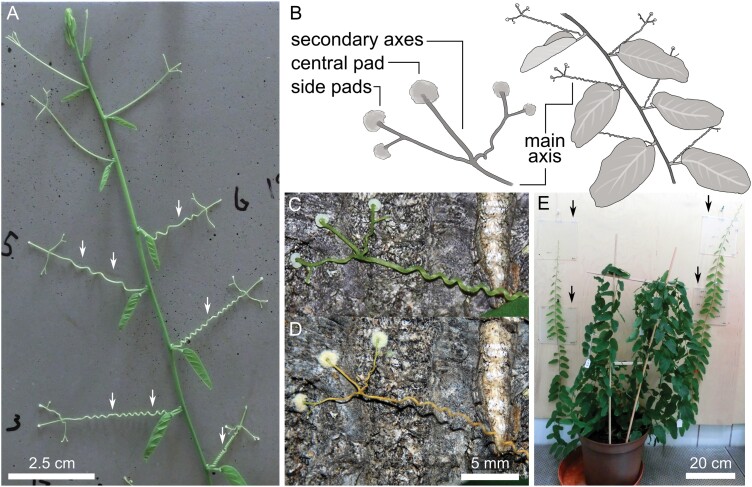
*P. discophora* habitus and tendril morphology. Tendrils originate from the alternating leaf axils. They contract spirally (free coiling) and form a spring-like structure, characterized by at least one ‘perversion’ (white arrows) (A). A tendril bears up to five pads and consists of the main axis basal to the furcation point and the secondary axes distal to the furcation point (B). During ontogeny, tendrils shift from the young green turgescent state (C) to a dried brownish died-off senescent state (D). Test plants were cultivated in a phytochamber and offered plates of various substrates (black arrows) for attachment; fixed to birch plywood (E).

Once attached to their support, virtually all tendrils contract spirally (termed free coiling) (Darwin, 1882; MacDougal, 1893, 1896) driven by the tendrils’ intrinsic curvature (McMillen and Goriely, 2002). Since the tendrils’ ends are fixed and thus are not free to rotate, the formation of at least two helices with opposite handedness, connected by one or multiple short straight portions referred to as ‘perversions’, is the optimal configuration from an energetic point of view (Darwin, 1882; MacDougal, 1893, 1896; McMillen and Goriely, 2002; Pieranski *et al.*, 2004) (Fig. 1A). This coiling movement both shortens the tendril, thereby pulling the stem closer to the support, and creates a spring-like structure that can absorb mechanical energy (Darwin, 1882; MacDougal, 1893; Putz and Holbrook, 1991). One phenomenon that can be observed when stretching tendrils of, for example, *Cucumis sativus* or *Echinocystis lobata*, is that, contrary to expectations, the tendril coils even further when pulled, adding turns on both sides of the perversion (overwinding), before it eventually starts unwinding when further elongated (Gerbode *et al.*, 2012) (see also Supplementary Videos S1 and S2 illustrating this behavior in *P. discophora*).

In contrast to climbing plants with tendrils performing contact coiling or with twining stems, those with adventitious roots or adhesive pads can also attach to and ascend on large diameter supports and even on flat surfaces (e.g. Putz, 1984). The convergent evolution of tendrils with adhesive pads is considered a special case that evolved in the Vitaceae, Passifloraceae, Bignoniaceae, and Cucurbitaceae (Sousa-Baena *et al.*, 2018). A prominent example of such a climber is Boston ivy (*Parthenocissus tricuspidata*, Vitaceae), the anatomy and attachment mechanics of whose tendrils have been thoroughly investigated by Steinbrecher *et al.* (2010, 2011). Its branched tendrils comprise a series of 5–10 secondary axes terminating in adhesive pads. The pads can individually withstand similarly high forces to those of the entire tendril–pad system. However, when the entire tendril–pad system is loaded, the system does not fail at once, but the pads fail in sequence, one or several at a time. This results in the energy dissipation of a tendril–pad system being considerably higher compared with that of an individual pad. Rather than being a force-optimized system, the attachment system is optimized towards withstanding deflections of the main axis and can be interpreted as a displacement-optimized fail-safe system.

The way in which adhesive pads are arranged along a tendril is highly variable between the species. Whereas juvenile plants of *Passiflora obovata* (Passifloraceae), for example, climb with unbranched tendrils (Krosnick *et al.*, 2013), many species forming adhesive pads have tendrils branched in various ways (Sousa-Baena *et al.*, 2018); bifid, trifid, and multifid tendrils with adhesive pads are found (de Wilde and Duyfjes, 1999; Seidelmann *et al.*, 2012; Lohmann and Taylor, 2014).

Climbing is widespread within the genus *Passiflora*, and the vast majority of its >500 species are climbers (Ulmer and MacDougal, 2004; Feuillet and MacDougal, 2007). Climbing *Passiflora* species bear axillary tendrils that are mostly simple and unbranched and that attach via contact coiling (Ulmer and MacDougal, 2004). However, in the section *Tryphostemmatoides*, tendrils with three to five branches are found that develop adhesive pads (Ulmer and MacDougal, 2004).

One example is *Passiflora discophora* P. Jørg. & Lawesson, a species that is endemic to the coastal Ecuadorean lowland and that grows in closed rainforest (Jørgensen *et al.*, 1987). On a flat substrate, its tendril tips respond to touch by callus-like growth into a hemispherical pad that penetrates even minute gaps and irregularities of the substrate, enabling optimal form closure supported by an extracellular adhesive substance (Bohn *et al.*, 2015). The pads are found at the apical end of the tendrils typically consisting of three secondary axes, two of which often further divide into a higher order of branching (Bohn *et al.*, 2015) (Fig. 1B). The mature tendril axis (Fig. 1C) consists of a central lignified tissue surrounded by parenchyma cells and of a band of sclerenchymatous cells (Bohn *et al.*, 2015). After ~2–6 weeks, the tendrils become senescent, and both pads and axes die off and desiccate (Fig. 1D) (Bohn *et al.*, 2015). With senescence, the parenchyma cells surrounding the lignified central part of the axes, plus many cells of the pad tissue, collapse (Bohn *et al.*, 2015). Nevertheless, the pads remain firmly attached to the substrate (Bohn *et al.*, 2015).

In this study, we complement the anatomical understanding of the adhesive pads of *P. discophora* as a crucial connection point between plant and substrate with an analysis of the adhesive systems’ mechanical performance. We measure how well these pads actually adhere by performing pull-off tests on individual pads. To quantify the rupture force on the level of the tendril system (axis and pads) and to find its ‘weakest link’, we also carried out pull-off tests on the entire tendrils. The influence of the substrate and of the tendrils’ ontogenetic stage on their performance is analyzed by comparing tendrils grown on substrates of different roughness and material, and in their different ontogenetic stages, namely in either the fully turgescent or senescent state.

## Materials and methods

### Plant cultivation

Potted *Passiflora discophora* P. Jørg. & Lawesson plants were cultivated in a phytochamber at temperatures of ~25 °C and 54% air humidity under a 12h light–12h dark cycle. Plant pots were positioned on the floor in the chamber, illuminated by HQI-E 400 W/D lamps (OSRAM, München, Germany), positioned at 2.50 m height. Plants were offered boards of birch plywood mounted on a scaffold as the basis of support on which to attach and climb (Fig. 1E). To investigate the influence of various attachment substrates, 15×15cm^2^ plates of a variety of substrate types were used. Negative silicone replicas (negative molds) were made from abrasive papers with a maximum particle size of 25.8 μm (P600; PRESI, Dortmund, Germany), 30 μm (3M-AO-30L, 3M Deutschland, Neuss, Germany), 58.8 μm (P240; PRESI), and 82 μm (P180; PRESI). These replicas were used as molds for epoxy resin (Epoxydharz/Härter HT2, Poxy Systems®, R&G Faserverbundwerkstoffe, Waldenbuch, Germany). For the production of the epoxy plates, we followed information from the manufacturer and used a mixing ratio of 100:48 according to the weight of resin to hardener. These replicas are referred to as ‘epoxy type25.8’, ‘epoxy type30’, ‘epoxy type58.8’, and ‘epoxy type82’ in the following. Additionally, plates of coarse mortar (maxit®-Zementmörtel-Mauermörtel, Saint-Gobain Weber, Düsseldorf, Germany), joint compound (Flexfuge, Obi Corporate Center, Wermelskirchen, Germany), and pieces of beech bark were used. Only fully differentiated tendrils (i.e. free coiling was terminated) were included in the analysis.

### Morphometric data

The pads and axes of the tendrils were photographed prior to and after the tests by using an Olympus stereo microscope (SZX9, OLYMPUS Deutschland, Hamburg, Germany). Measurements on the images were carried out using the software ImageJ 1.47v (National Institute of Health, Bethesda, MD, USA). For tests on entire tendrils, the number of intact pads per tendril was counted, and their projected pad areas were measured and summed to give the ‘total pad area per tendril’. A pad was defined as a tendril tip that had formed pad tissue and was in contact with the substrate. On coarse mortar, the pads commonly detached during development, taking with them grains of the substrate. These detached pads were measured nonetheless and included in the morphometric analyses, and the mean pad area per tendril was calculated. Further, the diameter of the tendril main axis was measured, and the ‘cross-sectional area of the main axis’ was calculated by assuming a circular cross-section (see Supplementary Fig. S1A). For a first-order estimation of the geometrical changes of the spring-like tendrils under loading, the axial length of tendrils in the coiled and in the straightened state was measured and the axial length straight/coiled ratio calculated (*n*_turgescent_=40, *n*_senescent_=57; see Supplementary Fig. S1B).

### Tensile tests

**Fig. 2.**
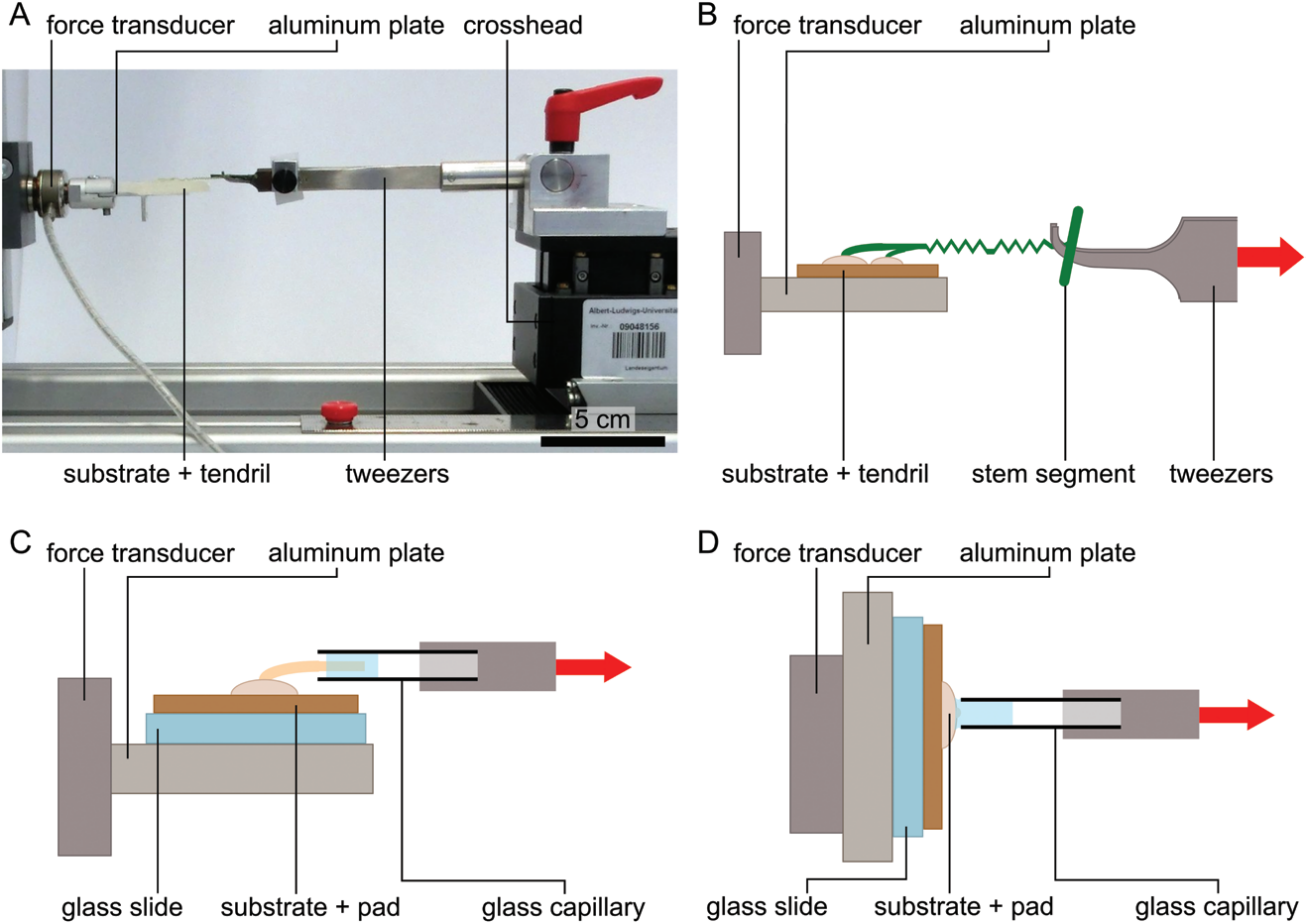
Experimental setup for tensile tests. For tensile tests on entire tendrils (setup 1), tendrils attached to various substrates were mounted via a small aluminum plate to the force transducer. The stem segment was hooked into tweezers connected to the crosshead (A and B). For individual pads subjected to a tensile force parallel to the substrate (setup 2a), the secondary axis was glued into a glass capillary connected to the crosshead (C). For individual pads subjected to a tensile force perpendicular to the substrate (setup 2b), the glass capillary was glued directly onto the pad (D). The direction of force application is indicated by a red arrow. Sketches not drawn to scale.

Tensile tests were carried out on entire tendrils with force application parallel to the substrate (setup 1) and on individual adhesive pads, which were tested both with force application parallel to the substrate (setup 2a) and with force application perpendicular to the substrate (setup 2b). A custom-built material testing device was used in these experiments (Fig. 2A); it consisted of an aluminum frame holding a 10N force transducer (SN949484, Honeywell, Columbus, OH, USA) and a micro step motor-controlled crosshead (ST4018M1804-KOWI1, Nanotec Electronic, Feldkirchen, Germany) that could be moved in the horizontal axis. The samples to be tested were mounted between the force transducer and the crosshead. Time, force, and displacement were recorded using the software LabVIEW™ (version 8.2, National Instruments Switzerland, Ennetbaden, Switzerland). For a detailed analysis of the failure behavior of the tendrils, all experiments were video-recorded (microscope camera USB 9.0 mio pixel with accompanying software eScope Version 1.1.7.17, Conrad Electronic SE, Hirschau, Germany).

In setup 1, entire tendrils (*n*_total_=149; *n*_turgescent_=47, *n*_senescent_=102) attached to each of the four types of epoxy resin plates, to the beech bark, or to the plywood were tested at a constant displacement velocity of 100 µm s^–1^. We were unable to test tendrils grown on coarse mortar and the joint compound, since the substrate failed during sample preparation for tensile tests. Turgescent tendrils grown on epoxy replicas were tested for only one roughness (epoxy type30). Samples were collected immediately prior to testing by carefully cutting a segment of the stem with one tendril and removing it together with the piece of substrate to which it had adhered. Cut surfaces were sealed with vaseline (Balea VASELINE parfümfrei, dm-drogerie Markt, Karlsruhe, Germany) to prevent dehydration. The substrates then were mounted on a small aluminum plate by superglue (UHU Sekundenkleber blitzschnell Pipette, UHU, Bühl, Germany). The aluminum plate was then fastened to the force transducer in a horizontal orientation at one end, and the stem segment was hooked into tweezers with bent tips connected to the crosshead at the other end (Fig. 2A, B). To facilitate sample handling, the following adjustment was introduced during tests with setup 1 and was used for all samples tested with setups 2 and 2a: a trimmed glass microscope slide was positioned between the substrate and aluminum plate and was fixed using epoxy glue (UHU Plus Sofortfest 2-K-Epoxidkleber, UHU, Bühl/Baden, Germany) to connect the substrate to the glass of the slide, and superglue to provide a safe connection between the glass of the slide and the aluminum plate. From the force–displacement data collected in tensile tests, we calculated energy dissipation as the area under the force–displacement curves.

In setups 2a and 2b, only senescent tendrils grown on plywood with a minimum number of three pads were used to allow a more standardized comparison between pads. If two side pads were present on one side, only the larger one was tested.

In setup 2a (Fig. 2C), individual adhesive pads (*n*=51) were tested with force application parallel to the substrate at a constant velocity of 1 µm s^–1^. Plywood sections with individual adhesive pads were mounted on a small aluminum plate (via a trimmed glass microscope slide as described above), fastened to the force transducer in a horizontal orientation. The secondary tendril axis of the adhesive pad was connected to the crosshead by attaching it with superglue (UHU Sekundenkleber blitzschnell Pipette) to a glass capillary (Borosilicate glass 3.3, Hilgenberg, Malsfeld, Germany). To improve curing of the superglue, the glass capillary was moistened with water vapor prior to and after gluing in the tendril axis, using an ultrasonic air-humidifier (custom-built apparatus). The glue was set to cure for 30min before the test was started.

In setup 2b (Fig. 2D), individual adhesive pads (*n*=35) were tested with force application perpendicular to the substrate, at a constant velocity of 1 µm s^–1^. As in setup 2a, plywood sections with adhesive pads were mounted on a small aluminum plate that was then fastened vertically to the force transducer. The secondary tendril axis was removed, and the adhesive pad was glued directly to the glass capillary by using superglue (moistened; see setup 2a). Direct contact between the capillary and adhesive pad was avoided to prevent injury of the pad. The curing time was set to 45min, as larger amounts of superglue were involved in this setup.

### Statistical analysis

Normal distribution and variance homogeneity were evaluated using the Shapiro–Wilk test and Levene’s test, respectively. Since, for each set of results, at least one comparison did not meet the assumptions for parametric testing, the following non-parametric tests were used. For the comparison of several groups, a Kruskal–Wallis test was employed followed by a post-hoc pairwise Wilcoxon rank-sum test with Holm’s correction for multiple testing, whereas for comparisons between two groups, a Wilcoxon rank-sum test was used. The following significance levels were applied: *P*-value ≥0.05, not significant (n.s.); *P*-value <0.05≥0.01, significant (∗); *P*-value <0.01≥0.001, high significant (∗∗); and *P*-value <0.001, highly significant (∗∗∗). All analyses were conducted using R (Version 3.6.2, R Core Team, 2019). As plants were held under close to uniform conditions during experimentation, and as almost all groups were sampled from two or three different ‘individuals’ (the individuality of test plants was unclear because of possible propagation by cut offshoots), we assumed independent samples, even though tendrils tested might have come from the genetically same plant and adhesive pads from the same tendril.

We are aware that not only growth conditions, but also factors such as age/size of the individual plant or shoot and its genetic disposition might influence tendril size. Nevertheless, we are convinced that the results and differences found when we compare the different groups here are robust.

## Results

### Force at failure of entire tendrils

**Fig. 3.**
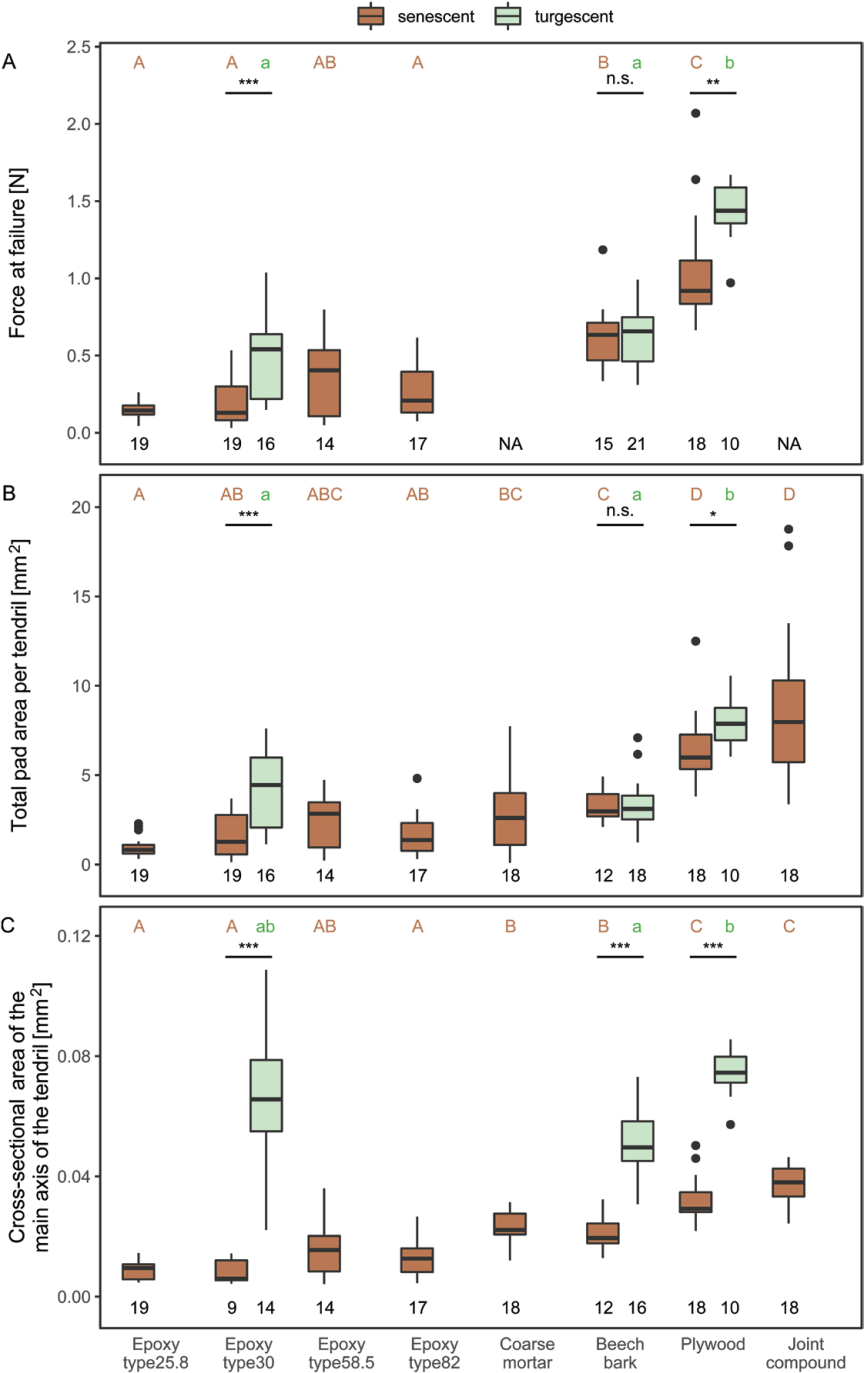
Mechanical and morphological characterization of tendrils grown on various substrates. Forces at failure for tendrils grown on epoxy substrates with different roughness, on beech bark and on plywood are shown for senescent (brown) and turgescent tendrils (green, only available for selected substrates) (A). Projected area of all pads per tendril, including substrates on which mechanical testing was not possible (NA) (B). Cross-sectional area of the main axis of the tendrils (C). All three variables differed significantly with substrate (Kruskal–Wallis test). Significantly (*P*<0.05) different groups are labeled with different letters (pairwise Wilcoxon post-hoc test with Holm correction). Forces at failure and total pad area for turgescent tendrils differed significantly from senescent tendrils on epoxy type30 and plywood, but not for beech bark substrate (Wilcoxon test). The cross-sectional area of the main axis of turgescent and senescent tendrils differed significantly on all three substrates (Wilcoxon test). Numbers indicate number of tested specimen.

Overall, the forces at failure of tested tendrils ranged between 0.03N and 2.07N for senescent tendrils (*n*=102) and between 0.15N and 1.67N for turgescent tendrils (*n*=47) (Fig. 3A). For senescent tendrils, the median force at failure was highest for tendrils grown on plywood substrate, lower on beech bark, and less than half as high on epoxy substrates. A significant difference was found in force at failure between substrates (Kruskal–Wallis test *P*=3×10^–12^; see Supplementary Table S1 for detailed test statistics). Forces at failure on plywood differed significantly from those on epoxy and beech bark. Forces at failure on beech bark differed from those on epoxy of all types, except for epoxy type58.5. The other pairwise comparisons showed no significant differences. Median force at failure was significantly lower for senescent tendrils than for turgescent tendrils on epoxy and on plywood (Wilcoxon test, *P*≤0.004). No significant difference was determined for tendrils grown on beech bark.

### Adhesive pad area

The number of pads per tendril varied between one and five (median=3). A frequency distribution of pad numbers is presented in Supplementary Fig. S2. Usually, one of the pads was considerably larger than the others. For senescent tendrils that were included in the mechanical analysis, the area of the largest pad per tendril ranged between 0.08mm^2^ and 4.31mm^2^ (*n*=99). For turgescent tendrils, this value ranged between 0.57mm^2^ and 3.75mm^2^ (*n*=46).

Median total pad area per tendril for senescent tendrils was highest on the joint compound and plywood (Fig. 3B). A significant difference in total pad area between substrates was detected (Kruskal–Wallis test, *P*=1×10^–15^). Significant differences were found for the same substrates that also differed in force at failure (Fig. 3A). On coarse mortar, the total pad area differed significantly from the total pad area on plywood, joint compound, or epoxy type25.8. On the joint compound, the area differed significantly from all substrates, except for plywood.

Median total pad area was lower for senescent tendrils than for turgescent tendrils on epoxy type30 and on plywood. The difference was significant (Wilcoxon test, *P*≤0.01). No significant difference was found on beech bark. A comparison of mean pad area per tendril (Supplementary Fig. S3) showed comparable tendencies with the comparison of the total pad area, except for the coarse mortar and the turgescent tendrils on beech bark.

### Cross-sectional area of the main axis of the tendrils

The cross-sectional area of the main axis of a tendril ranged between 0.004mm^2^ and 0.050mm^2^ for senescent tendrils (*n*=125) and between 0.02mm^2^ and 0.11mm^2^ for turgescent tendrils (*n*=40) (Fig. 3C).

For senescent tendrils, the median cross-sectional area was highest on the joint compound and plywood. A significant difference in cross-sectional area between substrates was observed (Kruskal–Wallis test, *P*<2×10^–16^). Significant differences were determined for the same substrate comparisons that also revealed differences in force at failure (Fig. 3A). On coarse mortar, the cross-sectional area significantly differed from all substrates, except for epoxy type58.5 and beech bark. On the joint compound, it differed significantly from all substrates, other than plywood.

The median cross-sectional area of the main axis was lower for senescent tendrils than for turgescent tendrils on epoxy type30, on plywood, and on beech bark. The difference was highly significant (Wilcoxon test, *P*≤8×10^–5^).

### Tendril failure behavior

**Fig. 4.**
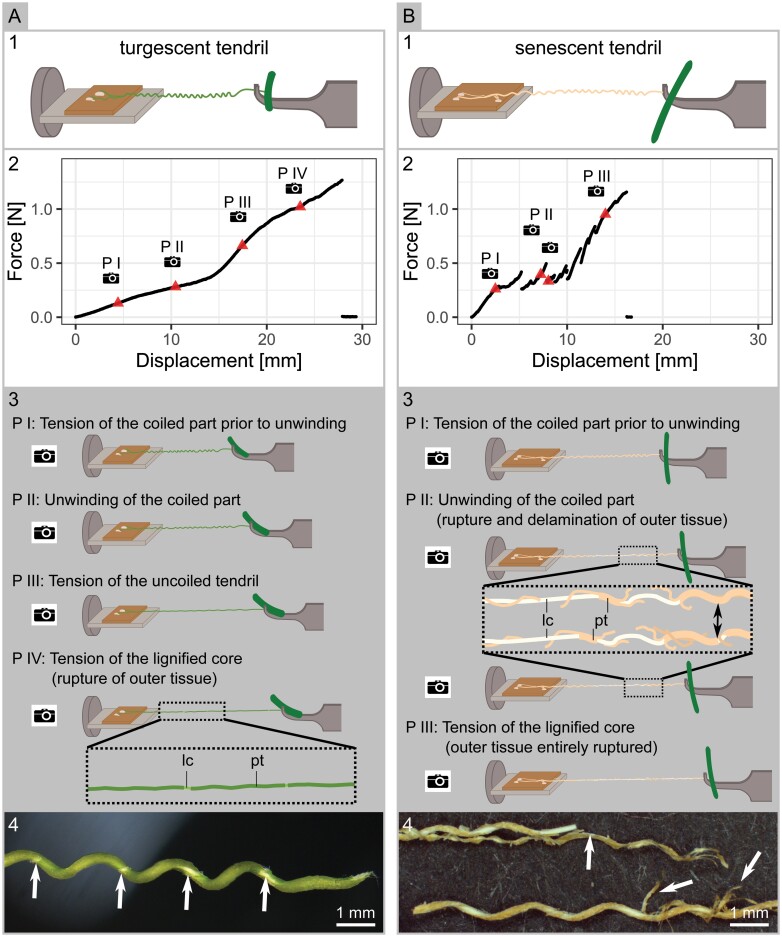
Tendril failure behavior. Tensile tests on turgescent (A) and senescent (B) tendrils (A.1/B.1). On the force–displacement curves, triangles mark the position where sketches of the tendril were redrawn from the videos (A.2/B.2). Sketches show the failure behavior of the tendrils under load that can be divided into four phases for turgescent tendrils and three phases for senescent tendrils. The sudden drops in force during phase II for senescent tendrils are associated with the rupturing of the outer tissue (black arrow) (A.3/B.3). Stereomicroscopic images after testing show the strong delamination of the outer dried parenchymatous tissue for senescent samples and various rupture points in the turgescent tissue (white arrows) (A.4/B.4). Abbreviations: lc, lignified core; pt, parenchymatous tissue. See Supplementary Videos S1 and S2 for corresponding video recordings.

During tensile tests, the coiled main axis of the tendrils became straightened. For almost all tendrils, this straightening was preceded by an overwinding before an unwinding of the coiled tendril axis started on increasing strain (see Supplementary Videos S1 and S2). The straightening led to a considerable increase in axial length, with a mean ratio of straightened length to coiled length of 1.4 (SD 0.2) for senescent tendrils and 1.8 (SD 0.4) for turgescent tendrils. Often, senescent tendrils grown on the epoxy replicates failed before being completely straightened (28 out of 69 tendrils). Otherwise, several characteristic phases in the failure behavior of the tendril main axis could be discerned, as illustrated in Fig. 4A and B and described in the following. Turgescent and senescent tendrils differed markedly in their behavior. Four and three distinct phases could be discerned, respectively (Fig. 4A.3, B.3). In phase I, both senescent and turgescent tendril coils became stretched, showing a spiral spring-like behavior and overwinding. Turgescent tendrils subsequently unwound, and the axis straightened along the axis of force application as it became further elongated (phase II), until it eventually reached a completely straight state (phase III). Finally, the parenchymatic tissue ruptured at various locations along the tendril (Fig. 4A.4), and the remaining lignified inner tissue (vascular cylinder) alone carried the load (phase IV). Finally, the main axis of the tendril failed abruptly.

Senescent tendrils differed from this behavior mainly in phase II (and to a lesser extent in phase III), which was characterized by distinct pre-failure events. The latter were evident from the video recordings (see Supplementary Videos S1 and S2), which revealed that parts of the dried outer parenchymatic tissue delaminated and ruptured during the stretching and unwinding of the tendril (Fig. 4B.4), thereby leaving only the lignified core to carry the load until eventual failure in phase III.

The shape of force–displacement curves was typically characterized by several linear regions of different slopes and, for senescent tendrils, by numerous smaller spikes indicating pre-failure events (Fig. 4A.2, B.2). The energy dissipated by the tendrils during loading as given by the area under the force–displacement curves ranged from 0.4 mJ to 6.4 mJ for senescent tendrils and from 3.6 mJ to 14.7 mJ for turgescent tendrils (median values from all substrates, see Supplementary Fig. S4).

In the turgescent tendrils, in particular, secondary axes also elongated visibly under load, and the furcation point usually became considerably displaced. The number and order of tendril elements that failed, the position of pads in relation to one another, and the degree of coiling of the secondary axes all varied. In the unloaded state, the central secondary axis was typically not coiled, whereas at least one of the two secondary side axes was usually coiled, but rarely with more than two windings.

Most tests ended in failure of only the main axis (74%), not accompanied by failure of other tendril elements, such as cohesive failure of the pads or failure of secondary axes. This failure mode was most frequent for both tendril conditions (turgescent or senescent) and on all substrates. The (successive) failure of one or several secondary axes was observed in 12% of tests, and a combination of failure of secondary axes and cohesive failure of pad(s) was recorded in 9% of the tests. Only rarely (in <5%) did a combined failure of the main axis and secondary axes, or cohesive failure of one or several pads without failure of other tendril elements, occur (see Supplementary Table S2 for detailed failure combinations).

When pads failed, some pad tissue always remained stuck to the substrate (i.e. no events of pure adhesive failure were observed). Moreover, none of the substrates included in the mechanical testing series failed. Relationships between force at failure, total pad area, and tendril cross-sectional area can thus be considered across substrates.

### Relationships between force at failure and tendril morphology

**Fig. 5.**
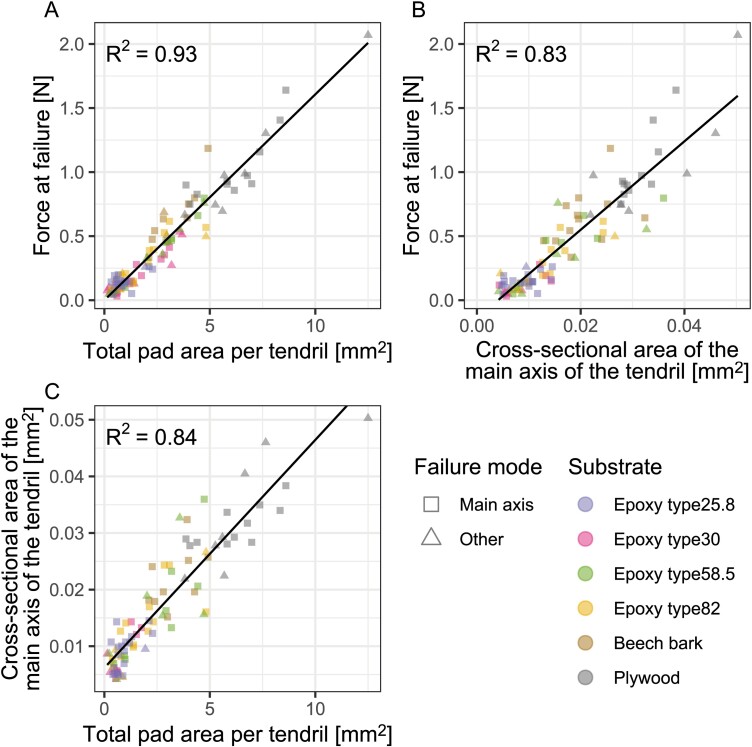
Relationships between the force at failure and morphological variables of the tendrils. Force at failure of senescent tendrils as a function of the total pad area (*n*=99) (A) and of the cross-sectional area of the main axis of the tendrils (*n*=89) (B). Relationship between the two morphological variables for tendrils tested mechanically (C). Exclusive failure of the main axis (squares) and other failure modes (triangles; see text for explanation) can be discriminated, as can the various substrates investigated. All linear regressions had high coefficients of determination (*R*^2^) and were significant with *P*<2×10^–16^.

For senescent tendrils, a linear relationship was observed between the force at failure and the total pad area and between the force at failure and the cross-sectional area of the tendril’s main axis (Fig. 5A, B). The same held true for the relationship between the two morphological variables of cross-sectional area of the main axis of the tendrils and the total pad area (Fig. 5C). The same positive linear correlations were found for turgescent tendrils (Supplementary Fig. S5), although the coefficients of determination were lower here.

### Adhesive pads

**Fig. 6.**
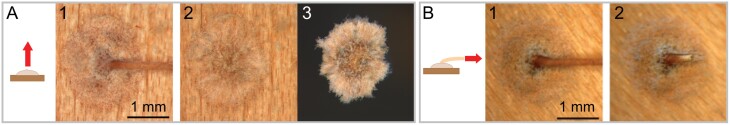
Failure behavior of individual adhesive pads. In senescent pads grown on a plywood substrate subjected to perpendicular loading (A.1, prior to testing), failure always occurred internally (A). The pulled-off pad itself ruptured and left remnants of the tissue on the substrate (A.2, remnants of tissue on the substrate; A.3, underside of pulled-off pad). Subjected to loading parallel to the substrate, the pad (B.1, prior to testing) failed either internally or at the secondary axis (B.2) (B).

Individual senescent pads grown on plywood were tested with the force applied either parallel or perpendicular to the substrate. When adhesive pads failed, pure adhesive failure never occurred. Instead, remains of pad tissue were always observed to persist on the substrate (Fig. 6A.2). In tests with force application parallel to the substrate, the secondary axis failed in 73% of tests (Fig. 6B), whereas the pad itself failed in 27% of tests.

**Fig. 7.**
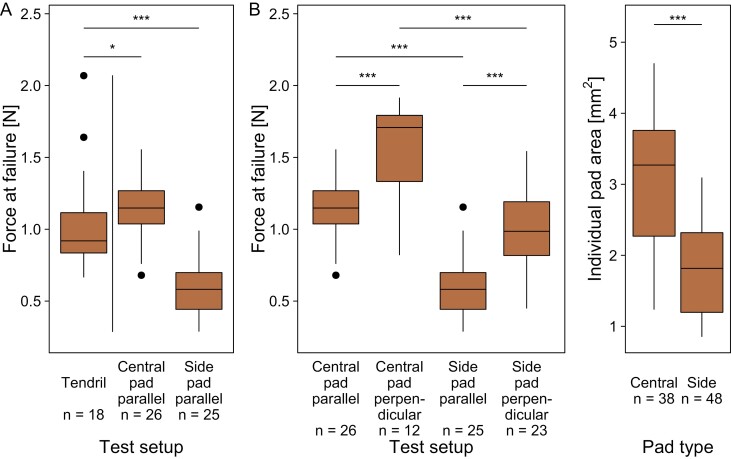
Force at failure of entire tendril–pad systems and of individual pads and areas of individual pads. Forces at failure for senescent tendril–pad systems grown on plywood (setup 1) compared with forces at failure measured for individual pads with a force application parallel to the substrate (setup 2, central and side pads) (A). Force at failure for central and side pads tested with force applications parallel and perpendicular to the substrate (B). Comparison of pad area for central and side pads (C). Significance levels from Wilcoxon tests are indicated above, based on Holm-corrected *P*-values for multiple testing in (A) and (B).

Tests on individual pads carried out with load application parallel to the substrate were compared with the results obtained for entire senescent tendrils grown on plywood (setup 1), since the orientation of applied force was comparable. In these cases, the median force at failure of the central pads was significantly higher than the force at failure of the entire tendril, whereas the median force at failure of the side pads was significantly smaller (Fig. 7A; see Supplementary Table S3 for test statistics).

For both central and side pads, the median force at failure was higher for pads tested with the force applied perpendicular to the substrate than with a force applied parallel to the substrate (Fig. 7B). In each test setup, the median force at failure was higher for central pads than for side pads, and the median area of central pads was significantly larger than that of side pads (Fig. 7C).

## Discussion

The presented experiments demonstrate that the adhesive pad-bearing tendrils of *P. discophora* provide a firm anchor to the substrate, despite the fact that their rupture force is merely 1.0±0.4N (entire senescent tendril on plywood, Fig. 3A) and thus is considerably smaller than values measured for other climbers featuring adhesive pads or adventitious roots: 3.8±2.4N for *Hedera helix* (2cm internodial piece with roots on bark), 18.3±6.0N for *Campsis radicans* (root cluster on wood), and 5.3±2.4N for *Parthenocissus tricuspidata* (lignified tendril on plaster) (Steinbrecher *et al.*, 2010). The difference from the last-mentioned, which is also a pad-bearing tendril climber, can be explained, because *P. discophora* bears markedly smaller pads than *P. tricuspidata* (individual pads with a mean projected area of 4.96±1.43mm^2^, Steinbrecher *et al.*, 2010). Even though plywood was the substrate on which *P. discophora* performed best and the difference from the rupture force of *P. tricuspidata* measured on plaster was obvious, we need to bear in mind that, at least for *P. discophora*, both pad size and tensile force are influenced by the substrate (Fig. 3).

**Fig. 8.**
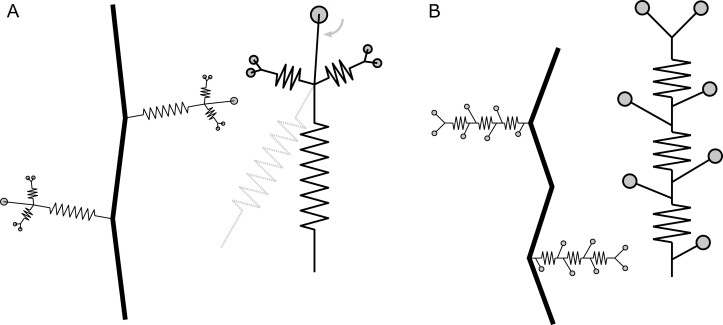
Attachment structures of *P. discophora* and *Parthenocissus tricuspidata*. Schematic representation of a tendril with pads and coiled axes and the arrangement of tendrils along a typical shoot of *P. discophora* (A) and *Parthenocissus tricuspidata* (B). Both tendrils can be viewed as a multianchored attachment system. The positioning of the attachment points differs: *P. discophora* exhibits a long spring-like main axis branching 3-fold. Deflection of the main axis and the expected resulting torsional shear loads are indicated in gray. In *Parthenocissus tricuspidata*, several pads are arranged along the main tendril axis. At the level of the shoot, both plants show an alternating positioning of attachment systems. Schematic in (B) reprinted from Steinbrecher *et al.* (2010). Quantifying the attachment strength of climbing plants: a new approach. Acta Biomaterialia **6,** 1497–1504, Copyright (2010), with permission from Elsevier.

At the level of the entire plant system, both plant species feature multiple adhesive tendril systems positioned alternately on the left and right side of a shoot (Fig. 8). Hereby, the self-weight of the plant and any additional endogenous (e.g. flowers or fruits) and exogenous (e.g. wind/rain loads, swaying supports, or climbing animals) loads inflicted on the plant body are supported by several tendrils simultaneously. The overall anchoring strength thus depends not only on the load that the individual tendril (or even an individual adhesive pad) can sustain before failing, but also on the ways that they fail and act together. The actual quantity (e.g. amount of force) and quality (direction of force application) of the load inflicted on a single tendril depends both on the weight of the plant (or part of the plant) and on the number of tendrils per shoot length. The force at failure of a single tendril is therefore not necessarily indicative of the strength of the anchoring of the plant as a whole to the substrate but allows an assessment and discussion of the form–structure–function relationship of the tendril–pad system and of its constituent parts (pads, and side and main tendril axes).

As the majority of tendrils failed at their main axis, the maximum force that they could sustain was usually not limited by the adhesive pads. The fact that the adhesive pads never failed at their interfaces but always within the pad tissue (i.e. cohesive failure, Fig. 6A) is in agreement with anatomical investigations showing a very tight form closure between the pad tissue and the substrate and also the presence of an extracellular substance most probably acting as adhesive (Bohn *et al.*, 2015). From the plant’s perspective, the formation of the interface to the substrate probably represents the most challenging part of the attachment process because it is ‘unpredictable’, since the plant cannot influence the properties of the substrate. We would therefore expect a high evolutionary pressure on the species towards evolving reliable physical and/or chemical adhesives suitable for a multitude of different substrates. Previous studies have shown a range of adequate adhesives comprising various groups of substances. The adhesive of *Parthenocissus quinquefolia* consists of rhamnogalacturonan I, callose, and other mucilaginous pectins (Bowling and Vaughn, 2008), whereas the adhesive secreted by the root hairs of English ivy is reported to consist of nanospherical arabinogalactan proteins, a hydroxyproline-rich glycoprotein (Huang *et al.*, 2019). The adhesive of *P. discophora* has not been chemically analyzed as yet but has been interpreted, based on various staining techniques, as being most probably composed of lipids and/or cutin (Bohn *et al.*, 2015).

We have been able to show that *P. discophora* can adhere to a wide variety of substrates (Fig. 3). With the epoxy surfaces of different roughness, we have observed no tendencies that would indicate an influence of roughness on the force at failure. However, changes of roughness over a different size range might have such effects.

Although the adhesive pads were able to bond to the epoxy surfaces, the adhesion was significantly better to the porous surfaces of plywood and beech bark (except for epoxy type58.5 to which adhesion was equally good as to beech bark, Fig. 3A). These substrates show the most similarities to the surfaces naturally occurring in the habitat of *P. discophora*, indicating that surface porosity might also influence the adhesive force. Cohesive failure of the substrate was not observed during our experiments, but coarse mortar substrate, for example, was too brittle to allow the non-destructive collection of samples for mechanical testing. Here, we commonly observed that pads started to develop but then later detached, carrying tiny pieces/grains of mortar with them. Such cohesive failure is likely to be a mode of failure for certain substrates on which *P. discophora* climbs in its natural habitat, for example loose rock or brittle tree bark. Indeed, exfoliating bark has been described as a defense mechanism of trees against epiphytes and climbing plants (e.g. ter Steege and Cornelissen, 1989; Zimmerman and Olmsted, 1992; López-Villalobos *et al*., 2008; Melzer *et al*., 2012). In the present study, the number of pads per tendril ranged from one up to of five, and seemed to depend on whether the tips at the end of the tendril branches were able to establish and to maintain contact with the supporting structure and eventually to grow into fully developed and functional pads. Some evidence has been found that young pads (loosely) adhering to the substrate can become detached again, as remains of detached and stunted pads and/or secondary axes are often visible at tendrils bearing fewer than five pads. Such detachment might result from premature (over)loading attributable to the increasing weight of the shoot (leaf growth) or because of external mechanical forces acting upon the plant or tendril or, as explained above, from cohesive failure of the substrate. One benefit of having a tendril with multiple adhesive pads might thus be to provide a system with back-up attachment points (see below for a discussion of fail-safe systems).

The risk of adhesion failure of the attachment structures is highest if peeling forces occur (Vogel, 1988). In the case of the pads of *P. discophora*, however, this risk seems to be minimized, as Bohn *et al.* (2015) have hypothesized based on the anatomy and morphology of the pads.

The force that the entire tendril–pad systems of *P. discophora* were able to sustain before failing varied significantly across tendrils grown on the various substrates (Fig. 3A). Since pure adhesive failure was not observed, these differences were an effect of the changes in size of the various parts rather than in adhesive strength. Our data suggest that the size of the projected pad area and the cross-section of the main axis scale linearly (thicker axes are equipped with a larger total projected pad area, Fig. 5C), which can be interpreted as a developmental ‘measure’ to prevent the introduction of ‘weak links’. Changes in both axis and pad sizes across substrates corresponded to differences in force at failure (Fig. 5A, B). Thus, no part of the tendril–pad system is extremely over- or undersized, even though we observed a clear tendency for the tendril main axis to be the weakest link under our test conditions. The finding that adhesive pads are slightly oversized suggests that they compensate for the ‘unpredictability’ of the substrate to which they attempt to adhere. Additionally, in contrast to the main tendril axis, which is typically loaded in an optimal collinear orientation (similar to a rope being pulled on), this is not necessarily the case for the adhesive pads, which might also face disadvantageous torsional shearing loads (Fig. 8A) under natural conditions.

The spring-like tendril morphology resulting from the free coiling of the tendril axis, composed of two helices with opposite handedness and connected by a perversion, represents a twistless spring (McMillen and Goriely, 2002). A normal spring fixed at both ends and without inversion of handedness and perversion would quickly stiffen because of its inability to untwist (Dai *et al.*, 2018). In contrast, a spring with a perversion can unwind and, in some cases, even overwind (Gerbode *et al.*, 2012; Dai *et al.*, 2018). We observed both of these winding motions in senescent and turgescent tendrils of *P. discophora*. Overall, the straightening of the coil resulted in considerable elongation, which explains the observed high energy dissipation until failure. Whereas turgescent tendrils were characterized by smooth force–displacement curves, the curve shape for senescent tendrils was clearly different (Fig. 4). When straightened by stretching and/or unwinding, senescent tendrils exhibited distinct pre-failure events, which were probably caused by the decreased elasticity of the dried-out senescent tissues. Dehydrated tissues typically have higher Young’s moduli than their hydrated counterparts (Niklas, 1992). As a consequence, the straightening of the senescent tendril not only leads to delamination of the dried and brittle outer parenchymatous tissues, but might also even damage the central lignified vascular cylinder. This explains the failure of senescent tendrils at lower forces than turgescent tendrils for two out of three sampled substrates (plywood and epoxy type30).

In addition to the change in the mechanical properties of the constituent materials of a plant organ, dehydration typically leads to geometric changes, notably shrinkage (Niklas, 1992). We have observed, in senescent tendrils, a strongly reduced cross-sectional area (Fig. 3C), which can mostly be attributed to the collapse of the parenchyma cells surrounding the lignified core. However, this change in diameter is unlikely to have a major influence on the rupture force since, even in the turgescent state, this parenchymatous tissue typically fails before the failure of the main axis of the tendril. Despite the decrease in force that they can withstand, the senescence of its tendrils is probably a beneficial trade-off for the plant overall, as a reliable anchorage is nevertheless maintained, while the tendril no longer needs to be supplied with water and nutrients, meaning that it no longer consumes metabolic energy.

Our experiments have shown that, within *P. discophora*’s tendril system (Fig. 8A), the central pads are the largest, and individually withstand higher forces than the entire tendril (Fig. 7). The combination of several pads therefore does not increase the critical load of the entire tendril, comparable with the findings in *P. tricuspidata* (Steinbrecher *et al*., 2010). In *P. tricuspidata*, the tendril system consists of several pads that are arranged along a coiled main tendril axis (Fig. 8B) and fail in sequence during loading, thereby increasing energy dissipation but not the detachment force of the system (Steinbrecher *et al.*, 2010). In *P. discophora*, the entire tendril most commonly fails at the main axis, and failure of the tendril attributable to failure of one or several individual pads is rare. Unlike in *P. tricuspidata*, the arrangement of several pads might thus not primarily serve to increase energy dissipation, but to provide back-up attachment points or to secure attachment at varying load angles (cf. Fig. 8A). Nevertheless we measured high energy dissipation in tensile tests on entire tendrils, which can be explained with the high elongations provided by the stretching and uncoiling of the spring-like coiled main tendril axis. Stretching of the secondary tendril side axes probably also contributes to the energy dissipation, albeit to a lesser extent. This comparison between *P. discophora* and *P. tricuspidata* strongly indicates that various means exist for building a fail-safe system in branched pad-bearing tendrils.

In *P. discophora*’s adhesive system, the central pad is larger and stronger than the side pads and is usually positioned more or less in alignment with the tendril main axis. The secondary axis (distal to the tendril branching) bearing the central pad is usually straight and not coiled and is therefore less extendable than the coiled side axes. This suggests that the central pads bear the highest fraction of the applied load. Whether the increased size of the central pad is a pre-determined morphological trait or the result of adaptive growth stimulated by the actual individual state of loading remains open. Such adaptations in plant growth, development, and composition as a response to mechanical stimulation have been shown for many plants and plant organs (e.g. Jaffe, 1973; Chehab *et al.*, 2009; Coutand, 2010). In addition to touch stimuli, which are known to initiate contact coiling of the tendrils of many plants (e.g. Jaffe and Galston, 1968; Engelberth *et al.*, 1995; Engelberth, 2003), experimental evidence has indicated that tension is a stimulus perceived by *Passiflora caerulea* tendrils (Brush, 1912).

The mechanical data on which this study is based were determined under precisely defined and controlled conditions to ensure comparability. Thus, entire tendrils were loaded parallel to the substrate and individual pads were loaded either parallel or perpendicular to the substrate. Under natural conditions, however, the loading situations might be much more variable in what concerns, for example, the angle of force application. Further studies should thus focus on varying load angles. We were able to show that the main axis plays a central (because limiting) role with respect to the breaking force of the adhesive system and that its mechanical properties are influenced by the ontogenetic stage. Future experiments should specifically target both mechanics and morphology of the isolated tendril main axis as this would allow a more in-depth analysis of the form–structure–function relationship of this particular spring-like structure. As to the effects of surface roughness, our results are limited to epoxy replicates of maximum particle size between 25.8 μm and 82 μm. Therefore, it would be worthwhile to investigate in further studies the influence of even smaller scale surface roughness, of surface chemistry, and especially of surface porosity.

## Conclusion

We have shown that the pad-bearing tendrils of *P. discophora* provide firm anchorage on supporting substrates of various materials and varying roughness by forming a multianchored attachment system with a total pad area that correlates with the tendril’s cross-sectional area. Both in turn correlate with the tendril’s breaking force. While the type of substrate significantly influences the force that the adhesive system can withstand, apparently by having an effect on tendril and pad morphology, we found no such apparent effect caused by controlled variation of the roughness of epoxy surfaces. We found that the tendril’s main axis rather than its pads or the pad–substrate interface are the weak link in the attachment system. This indicates that slight oversizing of pads allows the plant to cope with the ‘unpredictability’ of substrates. Moreover, a single large central pad loaded parallel to its secondary axis withstands higher forces before failing than the entire tendril does when loaded in parallel orientation. This suggests that the side pads do not increase the critical load of the entire tendril in this loading situation. With the high strain at rupture entailing a high energy dissipation of the spring-like tendril structure, the tendrils of *Passiflora discophora* can be regarded as fail-safe attachment systems. The tendril remains securely anchored, even when senescent and thus being independent of metabolic energy supply, rendering it a highly efficient system. The complex mechanical functioning of the individual tendril–pad system, the potential distribution of loads over a larger number of tendrils, and the ‘cost–benefit’ ratio of the system in the long term make plants featuring these tendril–pad attachment systems interesting study objects. Further work based on such biological problems can now be extended towards possible biomimetic applications, especially within the field of plant-inspired soft robotics and technical anchoring systems.

## Supplementary data

The following supplementary data are available at *JXB* online.

Table S1. Test statistics for data presented in Fig. 3.

Table S2. Failure combinations for tensile tests on entire tendrils.

Table S3. Test statistics for data presented in Fig. 7.

Fig. S1. Definition of morphological variables.

Fig. S2. Frequency distribution of pad number per tendril.

Fig. S3. Mean pad area per tendril for tendrils grown on various substrates.

Fig. S4. Energy dissipation for tendrils grown on various substrates.

Fig. S5. Relationships between force at failure and morphological variables of turgescent tendrils.

Video S1. Video recording of tensile test on an entire turgescent tendril (corresponding to Fig. 5A).

Video S2. Video recording of tensile test on an entire senescent tendril (corresponding to Fig. 5B).

erab456_suppl_Supplementary_Tables_S1-S3_Figures_S1-S5_Protocol_S1

erab456_suppl_Supplementary_Video_S1

erab456_suppl_Supplementary_Video_S2

## Data Availability

The data supporting the findings of this study are available from the corresponding author, Frederike Klimm, upon reasonable request.
