# Supplementary material for: Biomechanics of tendrils and adhesive pads of the climbing passion flower Passiflora discophora
Source: J Exp Bot. 2021 Oct 21;73(4):1190–203. doi: 10.1093/jxb/erab456 (PMC8866636; doi:10.1093/jxb/erab456)
Supplement: erab456_suppl_Supplementary_Tables_S1-S3_Figures_S1-S5_Protocol_S1 [file erab456_suppl_supplementary_tables_s1-s3_figures_s1-s5_protocol_s1.pdf]

Supplementary material

Table S1. Test statistics for data presented in Fig. 3. Descriptive statistics and results of Kruskal-Wallis tests and Wilcoxon tests for force at failure, total pad area, and cross-sectional area of the main axis of turgescent and senescent tendrils grown on the various substrates are listed below.

**Fig. 3A force at failure**						
	epoxy type25.8	epoxy type30	epoxy type58.5	epoxy type82	beech bark	plywood
median [N] senescent	0.14	0.13	0.40	0.21	0.63	0.92
IQR [N] senescent	0.06	0.22	0.43	0.26	0.24	0.28
median [N] turgescent	NA	0.54	NA	NA	0.66	1.44
IQR [N] turgescent	NA	0.42	NA	NA	0.29	0.23
Kruskal-Wallis test	senescent tendrils			turgescent tendrils		
	χ^2	df	p	χ^2	df	p
	62.8	5	$3\cdot10^{-12}$	25.0	2	$4\cdot10^{-6}$
p-values pairwise Wilcoxon post hoc test with Holm correction senescent tendrils						
	epoxy type25.8	epoxy type30	epoxy type58.5	epoxy type82	beech bark	
epoxy type30	1					
epoxy type58.5	0.4	0.4				
epoxy type82	0.4	0.5	1			
beech bark	$9\cdot10^{-6}$	$7\cdot10^{-6}$	0.2	0.001		
plywood	$3\cdot10^{-6}$	$3\cdot10^{-6}$	$8\cdot10^{-5}$	$6\cdot10^{-6}$	0.0007	
p-values pairwise Wilcoxon post hoc test with Holm correction turgescent tendrils						
	epoxy type30	beech bark				
beech bark	0.05					
plywood	$2\cdot10^{-6}$	$2\cdot10^{-5}$				
Wilcoxon test turgescent vs. senescent tendrils						
	W	p-value				
epoxy type 30	49	0.0004				
plywood	29	0.004				
beech bark	146	0.7				

**Fig. 3B total pad area**								
	epoxy type25.8	epoxy type30	epoxy type58.5	epoxy type82	beech bark	joint compound	coarse mortar	plywood
median [mm²] senescent	0.82	1.27	2.84	1.37	2.97	7.97	2.60	5.99
IQR [mm²] senescent	0.49	2.20	2.52	1.55	1.24	4.57	2.90	1.93
median [mm²] turgescent	NA	4.45	NA	NA	3.11	NA	NA	7.88
IQR [mm²] turgescent	NA	3.91	NA	NA	1.34	NA	NA	1.81

Kruskal-Wallis test	senescent tendrils				turgescent tendrils			
	χ^2	df	P		χ^2	df	p	
	84.8	7	$1\cdot10^{-15}$		19.4	2	$6\cdot10^{-5}$	
p-values pairwise Wilcoxon post hoc test with Holm correction senescent tendrils								
	epoxy type25.8	epoxy type30	epoxy type58.5	epoxy type82	beech bark	joint com-pound	coarse mortar	
epoxy type30	1							
epoxy type58.5	0.2	1						
epoxy type82	1	1	1					
beech bark	0.0001	0.03	1	0.048				
joint compound	$5\cdot10^{-6}$	$1\cdot10^{-8}$	$1\cdot10^{-5}$	$3\cdot10^{-7}$	$3\cdot10^{-5}$			
coarse mortar	0.005	0.4	1	0.6	1	$2\cdot10^{-5}$		
plywood	$5\cdot10^{-6}$	$3\cdot10^{-9}$	$2\cdot10^{-5}$	$7\cdot10^{-7}$	$8\cdot10^{-5}$	0.5	$7\cdot10^{-5}$	
p-values pairwise Wilcoxon post hoc test with Holm correction turgescent tendrils								
	epoxy type30	beech bark						
beech bark	0.3							
plywood	0.0002	$6\cdot10^{-6}$						
Wilcoxon test turgescent vs. senescent tendrils								
	W	p-value						
epoxy type 30	42	0.0001						
plywood	39	0.01						
beech bark	104	0.9						

**Fig. 3C cross-sectional area main axis**								
	epoxy type25.8	epoxy type30	epoxy type58.5	epoxy type82	beech bark	joint com-pound	coarse mortar	plywood
median [mm²] senescent	0.010	0.006	0.016	0.013	0.019	0.038	0.022	0.029
IQR [mm²] senescent	0.005	0.007	0.012	0.008	0.007	0.009	0.007	0.007
median [mm²] turgescent	NA	0.066	NA	NA	0.050	NA	NA	0.075
IQR [mm²] turgescent	NA	0.024	NA	NA	0.013	NA	NA	0.009
Kruskal-Wallis test	senescent tendrils				turgescent tendrils			
	χ^2	df	p		χ^2	df	p	
	91.4	7	$< 2\cdot10^{-16}$		13.6	2	0.001	
p-values pairwise Wilcoxon post hoc test with Holm correction senescent tendrils								
	epoxy type25.8	epoxy type30	epoxy type58.5	epoxy type82	beech bark	joint com-pound	coarse mortar	
epoxy type30	0.9							
epoxy type58.5	0.2	0.2						
epoxy type82	0.3	0.3	0.9					
beech bark	0.0001	0.003	0.4	0.04				
joint compound	$6\cdot10^{-6}$	0.0007	0.0003	$2\cdot10^{-5}$	0.0004			
coarse mortar	$9\cdot10^{-6}$	0.001	0.1	0.003	0.4	$7\cdot10^{-5}$		
plywood	$6\cdot10^{-6}$	0.001	0.003	$3\cdot10^{-5}$	0.002	0.2	0.003	

p-values pairwise Wilcoxon post hoc test with Holm correction turgescent tendrils								
	epoxy type30	beech bark						
beech bark	0.08							
plywood	0.2	0.0005						
**Wilcoxon test turgescent vs. senescent**								
	W	p-value						
epoxy type 30	0	$8\cdot10^{-5}$						
plywood	0	$2\cdot10^{-5}$						
beech bark	1.5	$1\cdot10^{-5}$						

Table S2. Failure combinations for tensile tests on entire tendrils. Observed failures are listed, sorted by the number of elements that (successively) ruptured during failure. The frequency for each number is given, as is a specification of the corresponding failed elements. Abbreviations: n: number of observations; ma: tendril main axis; sa: secondary tendril axis; p: adhesive pad.

	Nb. of failed elements	n (%)	Failed elements	n	Failed elements	n	Failed elements	n	Failed elements	n
**Turgescent tendrils (n = 47)**	1	39 (83%)	ma	39						
	2	3 (6%)	2x sa	2	2x p	1				
	3	3 (6%)	3x sa	1	2x sa, p	2				
	4	2 (4%)	2x sa, 2x p	1	sa, 3x p	1				
	5	0 (0%)								
**Senescent tendrils (n = 102)**	1	73 (72%)	ma	71	sa	1	p	1		
	2	12 (12%)	2x sa	6	sa, ma	4	2x p	1	sa, p	1
	3	13 (13%)	3x sa	7	2x sa, p	3	sa, 2x p	3		
	4	3 (3%)	2x sa, 2x p	1	4x sa	1	3x sa, p	1		
	5	1 (1%)	4x sa, 1x p							

Table S3. Test statistics for data presented in Fig. 7. Descriptive statistics and results of Wilcoxon tests are listed, corrected for multiple tests in (A) and (B) by Holm correction.

			tendril	central pad parallel	side pad parallel	central pad perpendicular	side pad perpendicular	central pad	side pad
**Fig. 7A**	force at failure [N]	median	0.92	1.15	0.58				
		IQR	0.28	0.23	0.26				
**Fig. 7B**	force at failure [N]	median		1.15	0.58	1.71	0.99		
		IQR		0.23	0.26	0.46	0.37		
**Fig. 7C**	individual pad area [mm²]	median						3.3	1.8
		IQR						1.5	1.1

		groups compared using Wilcoxon test		W	p-value
**Fig. 7A**	force at failure	tendril	central pad parallel	143	0.03
		tendril	side pad parallel	407	$2\cdot10^{-5}$
**Fig. 7B**	force at failure	central pad parallel	side pad parallel	626	$2\cdot10^{-10}$
		central pad perpendicular	side pad perpendicular	244	0.0002
		central pad parallel	central pad perpendicular	50	0.0005
		side pad parallel	side pad perpendicular	61	$1\cdot10^{-6}$
**Fig. 7C**	individual pad area	central pad	side pad	1504.5	$3\cdot10^{-7}$

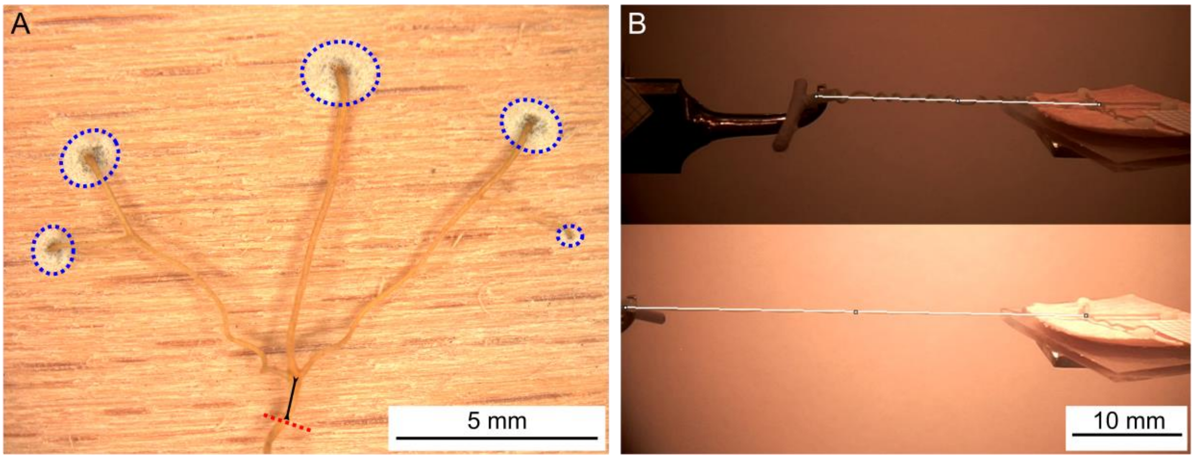

Fig. S1. Definition of morphological variables. The sum of the projected attachment area of all adhesive pads (dotted blue line) represents the "total pad area" per tendril. For the "cross-sectional area of the main axis of the tendril", the tendril diameter was measured at a distance of approx. 1 mm (black line) from the furcation point and the cross-sectional area was calculated by assuming a circular cross-section **(A)**. Length measurements for the coiled and straightened tendril (white) on stills from video recordings of the tensile test, from which the ratio of the axial length straight/coiled tendril was calculated **(B)**.

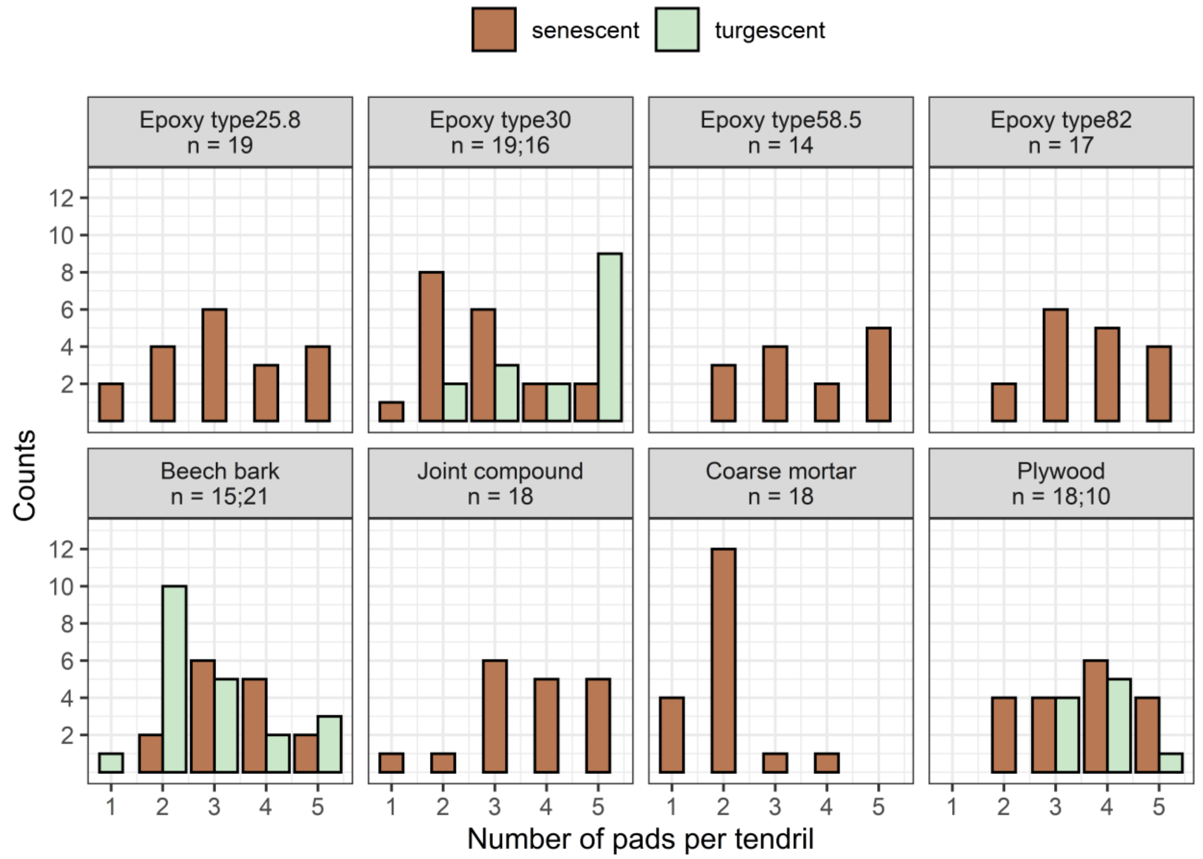

Fig. S2. Frequency distribution of pad number per tendril. The frequency of tendrils with pad numbers 1, 2, 3, 4 or 5 occurring in the sample is plotted for each substrate investigated, for senescent (brown) and turgescent (green) tendrils if available. No analysis of substrate effect on pad number was carried out, as sample sizes per substrate were small compared with the five possible categories observed (an analysis of pad geometry was not within the original scope of this work).

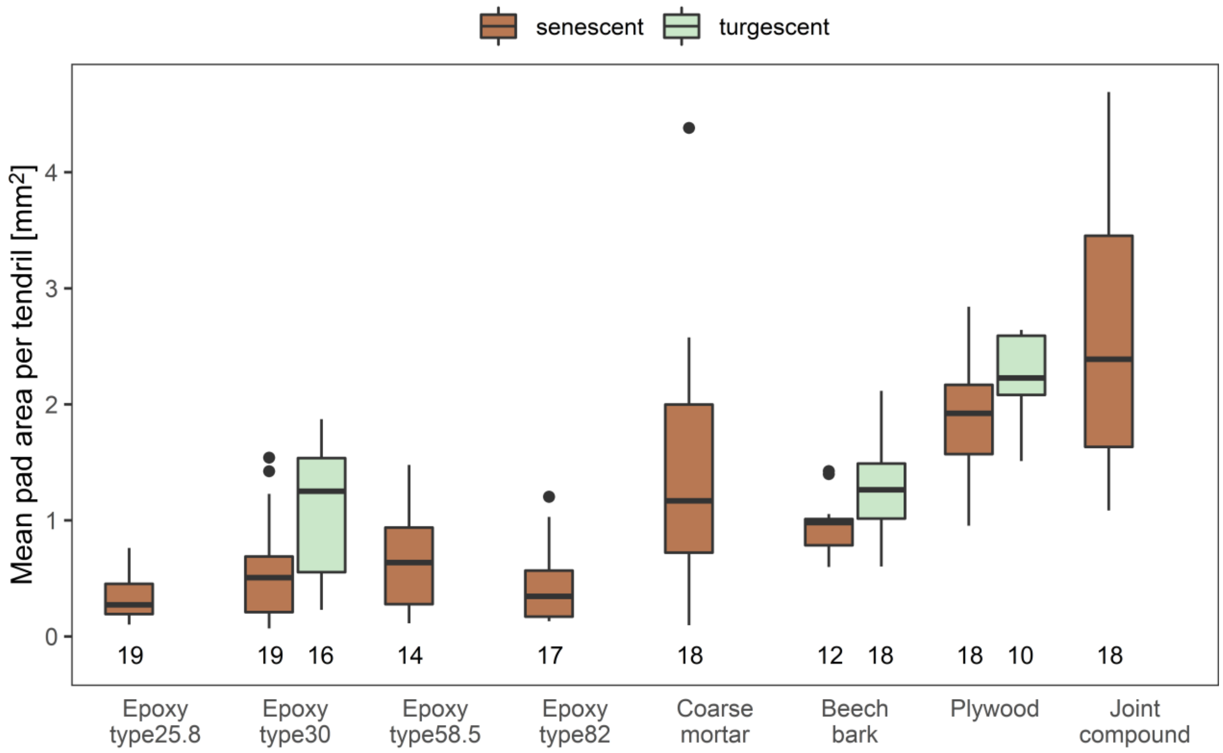

Fig. S3. Mean pad area per tendril for tendrils grown on various substrates. In addition to the values presented in Fig. 3, the summed area of all pads per tendril divided by the number of pads per tendril is presented. Results for senescent (brown) and turgescent (green, only available for selected substrates) tendrils grown on various substrates are shown, including substrates for which mechanical testing was not feasible.

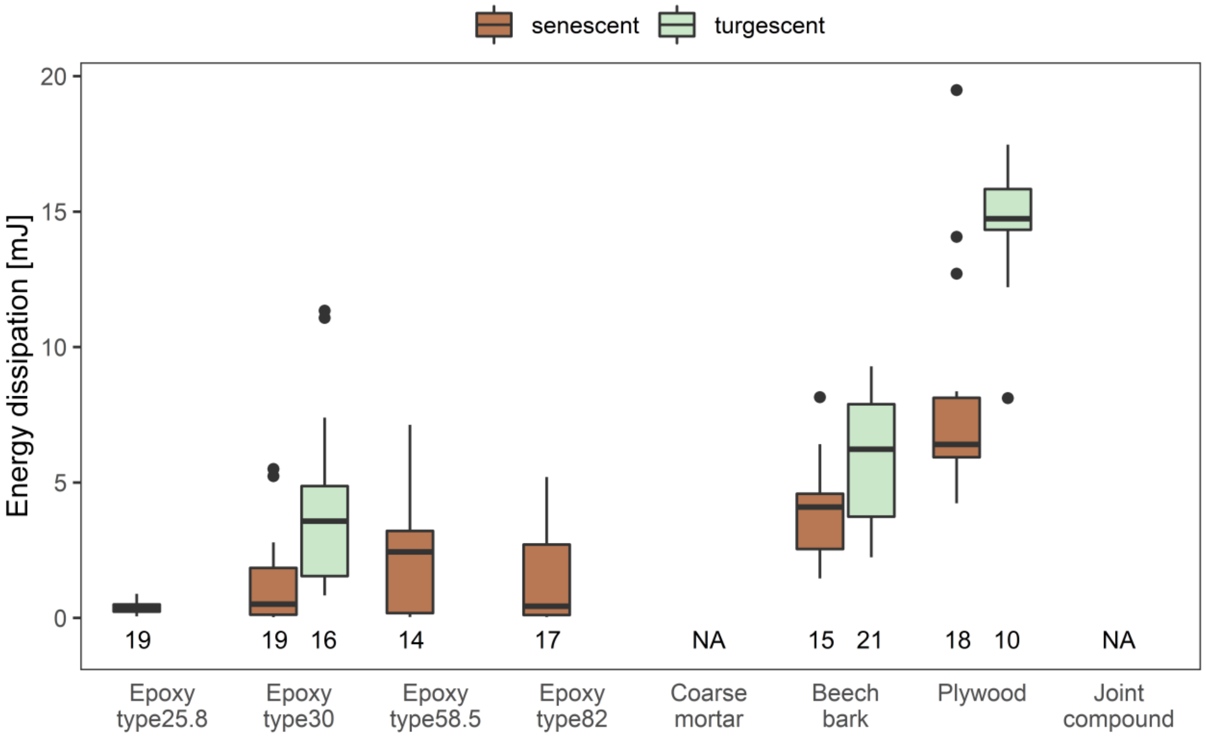

Fig. S4. Energy dissipation for tendrils grown on various substrates. Energy dissipation as derived from the area under the force-displacement curves measured in tensile tests on entire tendrils. Results for senescent (brown) and turgescent (green, only available for selected substrates) tendrils grown on various substrates are shown.

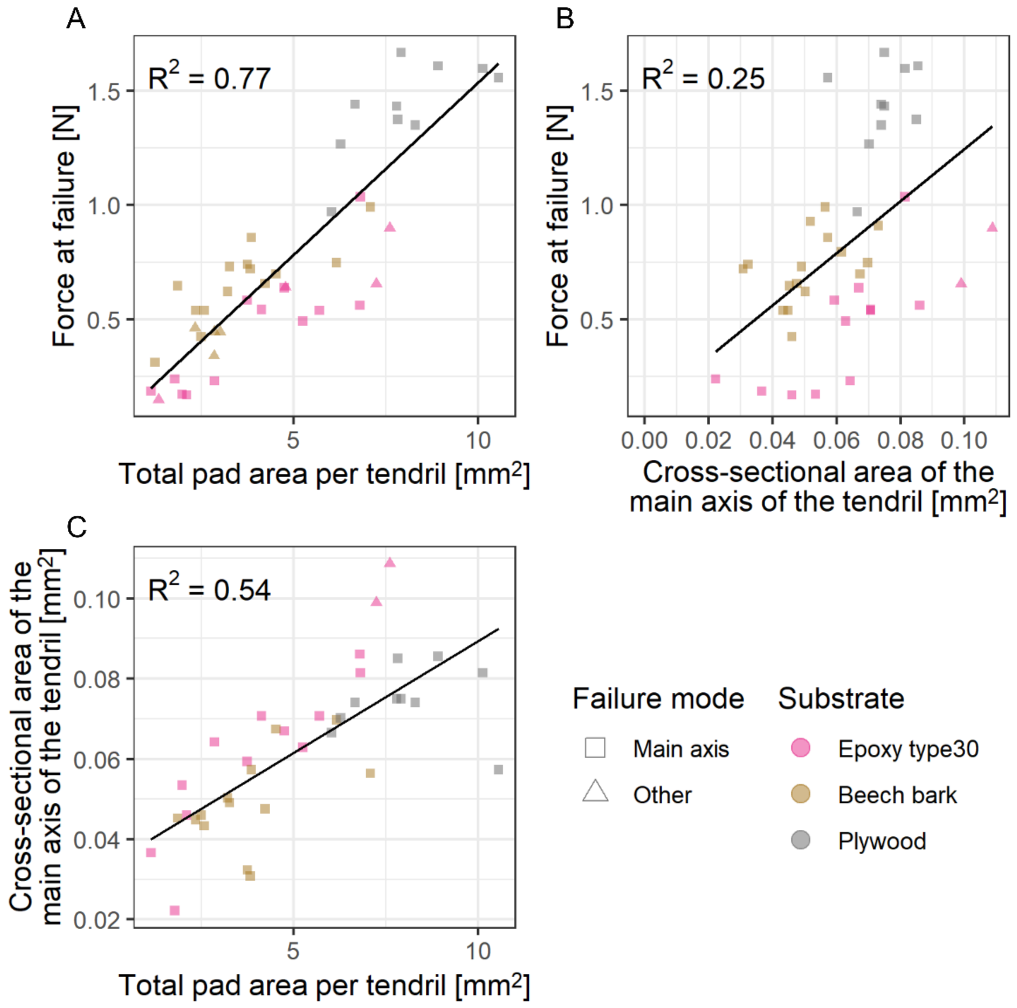

Fig. S5. Relationships between force at failure and morphological variables of turgescent tendrils. Force at failure of turgescent tendrils as a function of the summed area of all pads per tendril (total pad area, n = 44) **(A)** and of the cross-sectional area of the main axis of the tendrils (n = 40) **(B)**. Relationship between the two morphological variables for tendrils tested mechanically **(C)**. Exclusive failure of the main axis (squares) and other failure modes (triangles, see text for explanation) are discriminated, as are the different substrates investigated. All linear regressions were significant with $p = 8{\cdot}10^{-15}$ (force at failure as a function of total pad area), $p = 0.001$ (force at failure as a function of cross-sectional area of the main axis), and $p = 2{\cdot}10^{-7}$ (cross-sectional area of the main axis as a function of total pad area). Coefficients of determination (R^2) are shown in the plots.
